# Multilayer Antibacterial Hydrogel Wound Dressings Incorporated With Green Synthesized Silver Nanoparticles

**DOI:** 10.1002/ddr.70102

**Published:** 2025-05-08

**Authors:** Ali Alipour, Omid Nejati, Gökçen Yaşayan, Ayça Girgin, Buse Tuğba Zaman, Betül Giray, Okşan Karal‐Yılmaz, Sezgin Bakırdere, Ayça Bal‐Öztürk

**Affiliations:** ^1^ Department of Stem Cell and Tissue Engineering, Institute of Graduate Education İstinye University İstanbul Türkiye; ^2^ Department of Pharmaceutical Technology, Faculty of Pharmacy Yeditepe University İstanbul Türkiye; ^3^ Chemistry Department, Faculty of Art and Science Yıldız Technical University İstanbul Türkiye; ^4^ Department of Pharmaceutical Microbiology, Faculty of Pharmacy İstinye University İstanbul Türkiye; ^5^ Department of Chemical Engineering, Faculty of Engineering and Architecture İstanbul Beykent University, Sariyer İstanbul Turkey; ^6^ Turkish Academy of Sciences (TÜBA) Ankara Türkiye; ^7^ Department of Analytical Chemistry, Faculty of Pharmacy İstinye University İstanbul Türkiye; ^8^ Stem Cell and Tissue Engineering Application and Research Center (ISUKOK) İstinye University İstanbul Türkiye

**Keywords:** antibacterial activity, green synthesis, multilayer wound dressing, silver nanoparticles, wound healing

## Abstract

Multilayer antibacterial hydrogel wound dressings were fabricated and characterized for wound healing applications. Dressings are designed to achieve infection control, moisture management in the wound area and to support wound healing. Multilayer wound dressings were prepared as three layers by solvent casting method. The upper layer is composed of kappa carrageenan and green synthesized silver nanoparticles (AgNPs, ~122 nm in size, zeta potential of –35 mV) to provide the moist control, and to form a barrier against microorganism attack. Lidocaine HCl loaded polyvinyl alcohol and chitosan‐based middle layer was designed to achieve controlled drug release and to add strength to the hydrogel structure. The lower layer is composed of hyaluronic acid and ovalbumin to serve a controlling membrane for controlled drug release, and to further support wound healing. Different amounts of AgNPs were used in formulations to evaluate their impact on multilayer wound dressings. The incorporation of AgNPs resulted in reduced swelling values and degradation rates of the multilayer wound dressings, enhanced mechanical capabilities, and no significant change in water vapor permeability values. They have demonstrated enhanced antibacterial efficacy against *Klebsiella pneumoniae*, *Bacillus subtilis* and *Candida albicans*. The optimal multilayered hydrogel, incorporating AgNPs and loaded with lidocaine HCl, has shown biocompatibility and hemocompatibility, exhibiting 60% degradation by day 14, water vapor permeability of 2022 ± 460 g/m^2^ over 24 h, a tensile strength of 6.71 ± 0.62 MPa, 36.38% ± 3.62% elongation at break, and 65.72% ± 14.80% drug release within 10 h, making it a promising candidate for facilitating the wound healing process.

## 1 Introduction

Skin is an enormous organ in our body, and performs numerous critical roles, including protecting the body from external injury, regulating body temperature, and repelling dangerous microorganisms. It provides a great potential for medical therapies, pharmaceutical and cosmetic applications (Geraili et al. 2018; Savoji et al. 2018; Sutterby et al. 2020). Because of the skin's critical roles, any injuries to it are of significant medical and scientific interest.

Skin injuries and burns are all widespread in everyday life (Gao et al. 2021). Skin and subcutaneous diseases are a large worldwide burden, that accounting for the 18th main cause of disability‐adjusted life year (DALY) worldwide, and the 4th most common cause of nonfatal burden in Global Burden of Disease (GBD) 2013 (Sutterby et al. 2020; Karimkhani et al. 2017). In spite of the fact that the skin has a heightened ability for renewal, skin attachments are difficult to restore under natural settings (Gu et al. 2021). Wound dressings are any coverings that come into close contact with the wound, providing protection against potential complications and fostering healing (Frykberg and Banks 2015; Rezvani Ghomi et al. 2023; Sood et al. 2014). Wound dressings also could help mitigate the crapshoot of disease while easing pain, providing condensation, stabilizing injured zones, maintaining the wound, and stimulating expedited rehab (Vermeulen. 2009; Wicker and Dalby 2016). Ideally, they should be biocompatible, have efficient moisture retention and mechanical resilience, and contain appropriate surface texture and biochemical attributes that promote cellular attachment, growth, and diversification. Ideally they should demonstrate antibacterial and antimicrobial properties for efficient treatment of infections (Zhang et al. 2023; Gounden and Singh 2024).

In recent years, significant attempts have been undertaken for fabrication of the wound dressings that support wound treatment. Hydrogels are one of the mostly studied topic in design of novel wound dressings due to their mild processing conditions and creating a moist environment that supports healing. Hydrogels possess a three‐dimensional (3D) hydrophilic network that closely mimics the microarchitecture of the extracellular matrix (ECM). Due to their unique and beneficial properties, hydrogels are increasingly being employed as wound dressings, particularly in the treatment of chronic and non‐healing wounds. These materials can be engineered to maintain optimal moisture levels, provide adequate oxygen permeability, and offer excellent flexibility and mechanical strength—key features that support effective wound healing (Zhang et al. 2023; Fan et al. 2021; Nuutila and Eriksson 2021; Yaşayan. 2022). Hydrogel networks can be formed through chemical and/or physical crosslinking. Chemically crosslinked hydrogels tend to offer greater structural stability, while physically crosslinked hydrogels are often considered safer for in vivo applications. Their properties can be finely tuned to maximize therapeutic outcomes, such as by enhancing drug delivery or modulating degradation rates. Due to their high‐water retention and swelling capacity, hydrogels help maintain a moist wound environment, reducing patient discomfort and promoting healing. In addition, their flexibility and mechanical resilience allow them to act as a protective barrier during the wound recovery process. Moreover, hydrogels serve as effective carriers for therapeutic agents and bioactive compounds, further accelerating tissue regeneration and wound repair (Zhang et al. 2023; Fan et al. 2021; Farahani and Shafiee 2021; Khan et al. 2021; Yang et al. 2021).

To meet the need for ideal wound dressings, in this study, antibacterial multilayer hydrogel wound dressings were developed for the infection control, moisture management and wound healing. In this 3‐layer wound dressing, the upper layer is composed of kappa carrageenan (K‐ca) and green synthesized silver nanoparticles (AgNPs) to provide the moist control, and to function as a barrier to microorganism attack. Different amount of AgNPs were used in formulations to see the effect of final properties of multilayer wound dressings. Lidocaine HCl loaded polyvinyl alcohol (PVA) and chitosan (CS)‐based middle layer was designed as a layer capable of controlled drug release and to add strength to the structure. The lower layer is composed of hyaluronic acid (HA) and ovalbumin (OVA) to serve a controlling membrane for controlled drug release and to further support wound healing. Multilayer hydrogel wound dressings were characterized using FTIR, and their swelling, degradation, and physical properties were evaluated in PBS (pH: 7.4). Mechanical strength, water vapor permeability, and in vitro drug release were assessed. Cytotoxicity and wound healing potential were tested via MTT and scratch assays on NIH/3T3 cells. Hemocompatibility and antibacterial activity against *Klebsiella pneumoniae, Candida albicans*, and *Bacillus subtilis* were also examined. This multifunctional design, combining natural polymers, AgNPs, and a local anesthetic, presents a promising platform for wound healing.

## 2 Materials and Methods

### 2.1 Materials

Hyaluronic acid (HA), 3‐[4, 5‐dimethylthiazol‐2‐yl]‐2,5 diphenyltetrazolium bromide (MTT), dimethyl sulfoxide (DMSO), silver nitrate (AgNO_3_), sodium hydroxide (NaOH), borate‐HCl buffer solution, kappa‐carrageenan (K‐ca), chitosan (CS), gelatin, poly(vinyl alcohol) (PVA), sodium sulfate (Na_2_SO_4_), acrylamide/bis‐acrylamide, Tris‐HCl, sodium dodecyl sulfate (SDS), ammonium persulfate (APS), N,N,N’,N’‐tetramethyl ethylenediamine (TEMED), 1‐ethyl‐3‐ (3‐dimethylaminopropyl) carbodiimide hydrochloride (EDC) and lidocaine HCl were purchased from Sigma Aldrich, Germany. Phosphate buffered saline (PBS) was purchased from Capricorn Scientific, Germany. Dulbecco's modified eagle medium (DMEM), fetal bovine serum (FBS) and penicillin/streptomycin (P/S) were bought from PAN‐Biotech, Biological Industries and ThermoFisher Scientific, respectively. Glycerol (99%) was purchased from TEKKIM, Türkiye. NIH/3T3 cells were purchased from ATCC, USA. All chemicals/reagents used throughout this study were of the analytical grade. Ultrapure water was produced from ELGA PureFlex III water purification system for sample preparation, dilution, and cleaning processes. All the other chemicals and reagents were purchased from Sigma unless otherwise specified.

### 2.2 Synthesis and Characterization Studies of Silver Nanoparticles

Silver nanoparticles (AgNPs) were biosynthesized using a biogenic method, following our previous study (Nejati. 2024). Apricot kernel skin extract served as both a green reducing agent for silver ions and a stabilizing agent. Dynamic light scattering (DLS) and zeta potential measurements of the biosynthesized AgNPs were conducted at room temperature using a Zetasizer Nano ZS (Malvern Panalytical, UK) to ascertain the particle size distribution and surface charge of the biosynthesized AgNPs, which are essential characteristics for assessing their stability and colloidal behavior.

### 2.3 Synthesis of Ovalbumin and Polyacrylamide Gel Electrophoresis

To synthesize ovalbumin (OVA), a 36% (w/v) aqueous solution of Na_2_SO_4_ was prepared and mixed with 200 mL of egg white. The resulting mixture was filtered through filter paper, and subsequently dialyzed against distilled water for 7 days using a 3500 Da dialysis membrane (MWCO). Distilled water was replaced three times per day. The solution was filtered again through filter paper, then freeze‐dried using a freeze‐dryer (Alpha 1‐2 LD plus) for 24 h (Liu et al. 2020). The final product was kept in the refrigerator.

SDS‐PAGE (sodium dodecyl sulphate‐polyacrylamide gel electrophoresis) was conducted to confirm the synthesis and evaluate the molecular integrity of OVA. For this purpose, separating gel (6%) and stacking gel (5%) was prepared. Precipitated samples were collected from −20°C, allowed to defrost at room temperature before being placed in a 95°C heat block. The samples were boiled 100°C for 10 min for denaturation of the proteins. Electrophoresis was carried out at 120 V, and the bromophenol blue dye was used to stain the proteins (Shojaee et al. 2015).

### 2.4 Preparation of Multilayer Hydrogel Wound Dressing Films

Multilayer hydrogel wound dressing films were fabricated utilizing the solvent casting technique owing to its simplicity, cost‐efficiency, and capability to yield consistent film structures. To prepare the top layer of the multilayer wound dressing films, K‐ca was firstly dissolved in water, and then AgNPs were added to the solution. After stirring at 37°C for 1 h, glycerol and the crosslinking agent CaCl_2_ were added to the solution. Different amounts of AgNPs were used in fabrication of the dressings, as 10, 20, 30, and 40 μg. The three‐layered hydrogels were named depending to the amount of AgNPs in the dressing; as 3L‐0, 3L‐10, 3L‐20 and 3L‐40, containing 0, 10, 20, 30, and 40 μg of AgNPs respectively. In preformulation studies, some of the studies carried only at the top layer of the dressing. Single‐layered samples are similarly named depending to the amount of AgNPs in the dressing as 1L‐0, 1L‐10, 1L‐20 and 1L‐40. (Table 1).

**Table 1:** Preparation procedure of multilayer hydrogel wound dressing film.

	Top layer	Middle layer	Bottom layer
**K‐ca**	0.15 g	—	—
**Glycerol**	0.34 g	—	—
**CaCl**_**2**_ **solution (0.8 g/mL)**	150 μL	—	—
**AgNPs**	10‐20‐30‐40 μg	—	—
**PVA solution (5%, w/v)**	—	4.5 g	—
**CS solution (1%, w/v)**	—	3 g	—
**Mixture of HA (1%, w/v) solution and OVA (2%, w/v) solution**	—	—	3.75 g
**Gelatin solution (10%, w/v)**	—	—	3.75 g
**EDC solution (20 mg/mL)**	—	—	75 µL

For the preparation of middle layer of multilayer wound dressing film, PVA (5%, w/v) and CS (1%, w/v) solutions were mixed at 37°C for 1 h (Table 1).

For the preparation of bottom layer of multilayer wound dressing film, mixture of 1% (w/v) HA solution and 2% (w/v) OVA solution were prepared by mixing the given amounts of ingredients at 37°C for 20 min. Then 10% (w/v) gelatin solution was added on this mixture. After mixing for a short time, EDC solution was added to the mixture (Table 1).

### 2.5 Characterization of Multilayer Hydrogel Wound Dressing Films

#### 2.5.1 FTIR Analysis

FTIR analysis were carried out using a FT/IR‐4600 (Jasco Inc., Japan) equipped with an ATR adapter at a wavelength range of 400–4000 cm^−1^ to identify the functional groups and confirm the chemical composition of the multilayer wound dressings.

#### 2.5.2 Swelling Tests

The swelling properties of the multilayer wound dressing films were examined in a PBS solution (pH: 7.4) at a temperature of 37°C to assess their fluid absorption ability, which is essential for sustaining a moist wound environment and efficient exudate management. Before analysis, the dry films were weighted (W_d_) and immersed in the PBS solution for 24 h. At various time intervals, any excess water on the swollen samples was removed by using a filter paper, and weighed immediately (W_s_). The swelling percentage for each sample was calculated using the following formula, where W_s_ represents the weight of the swollen film samples, and W_d_ denotes the weight of the dry film samples (Goetsch and Niesler 2011; Yaşayan et al. 2021):

$$\text{Swelling}\;[\%]=\frac{\left(W_{s}-W_{d}\right)}{W_{d}}\times 100$$

#### 2.5.3 Hydrolytic Degradation Properties of Wound Dressings

The degradation characteristics of the multilayer wound dressing films were evaluated in a PBS solution (pH: 7.4) at 37°C over a period of 14 days to determine their structural integrity and estimate their in vivo longevity under physiological settings. Wound dressing samples in equal dimensions completely dried and weighed. They were then immersed in a PBS solution (pH: 7.4) at a temperature of 37°C. After 21 days, the samples were taken out, freeze‐dried, and the decrease in weight was calculated using the following formula, where W_before_ represents the weight of the dry samples before hydrolytic degradation, and W_after_ denotes the weight following hydrolytic degradation (Goetsch and Niesler 2011; Yaşayan et al. 2021):

$$\text{Weight remaining}\;[\%]=\frac{W_{\text{after}}}{W_{\text{before}}}\times 100$$

#### 2.5.4 Physical Properties and pH Determination

Film thickness and pH measurements were conducted to assess the physical homogeneity and surface acidity of the multilayer wound dressing films, since these factors are essential for guaranteeing optimal mechanical performance, ease of application, and compatibility with the wound environment. An OMS BX1 thickness gauge was used to measure thickness of the samples. The average and standard deviation from a minimum of three distinct locations, measured with an accuracy of 0.001 mm were provided (Türkoğlu et al. 2021). For determination of the pH of the samples, small pieces of samples, weighing 0.5 ± 0.001 g, were cut. The samples were placed in conical tubes, and mixed with ultrapure water at a 1:100 ratio. These tubes were then incubated at 37°C ± 1°C. A Hanna Instruments H221 pH meter (with a sensitivity of 0.01) was used to measure the pH at 2‐h and 24‐h intervals. The average and standard deviation from a minimum of five measurements were presented (Türkoğlu et al. 2021).

#### 2.5.5 Determination of Water Vapor Permeability

The water vapor permeability of the hydrogel wound dressing films was assessed by adapting the ASTM E96‐00 standard test protocols (Wang et al. 2014) for material water vapor transmission to assess their capacity to sustain an appropriate moisture equilibrium at the wound site, which is crucial for efficient wound healing. A solvent‐based adhesive was employed to secure the sample onto a glass vial (diameter: 12.5 mm) filled with deionized water. The vial was subsequently placed in an incubator at 34°C ± 2°C and a relative humidity of 35% ± 2% (Türkoğlu et al. 2021; Wang et al. 2014). Water vapor transmission rate (WVTR) of at least three samples at skin temperature was computed by measuring the weight loss after 24 h using equation below, and is presented in grams per square meter per day (g/m^2^/day). ΔW/Δt is the amount of water gain per unit time. A is area exposed to water surface in m^2^. Before the assessment, the specimens were stored in a controlled environment with a temperature of 20°C and a relative humidity of 65% ± 4% (Türkoğlu et al. 2021; Wang et al. 2014; Bajpai et al. 2014).

$$\text{WVTR}\;(g/m2/\text{day})=\frac{\Delta W}{\Delta \text{t x A}}\;$$

#### 2.5.6 Mechanical Test

Tensile tests were conducted on the multilayer hydrogel films to assess their strength and elasticity, essential for assuring durability and flexibility in their application as wound dressings. Mechanical properties of the hydrogel wound dressing films were evaluated using a Stable Micro Systems Texture Analyser (TA. XT Express, UK) fitted with a 10 kg load cell. The film samples were cut to a size of 20 x 10 mm to ensure sample uniformity. The mechanical strength tests were conducted at a constant tensile rate of 10 mm/min on a minimum of three samples (Bal‐Öztürk 2024a, 2024b).

#### 2.5.7 Drug Loading and Drug Release Tests

Lidocaine HCl was loaded into the middle‐layer of the multilayer hydrogel wound dressing films. During the preparation of the middle layer, 20 mg of lidocaine HCl was added to the solution given in Table 1. Lidocaine HCl release studies were performed from both mono‐ and multilayer‐ hydrogels. The study was conducted under sink conditions. Hydrogels with known weights were immersed in a determined amount of PBS solution at 37°C. At certain time intervals, samples were taken from the test solutions and the same volume of fresh PBS solution was added to the media. The drug amounts were determined using a UV‐Vis spectrophotometer at 203 nm.

#### 2.5.8 MTT Test

Cytotoxicity studies on hydrogel wound dressing films were evaluated using an indirect MTT assay on NIH/3T3 cell lines. NIH/3T3 cells were cultured in DMEM supplemented with 10% FBS, 1% l‐glutamine and 1.0% (v/v) P/S at 37°C in a humid atmosphere with 5% CO_2_. The media replenished in every 3 days. The cells were isolated from the flask at 80% density using a 0.02% EDTA/0.05 trypsin solution. They were then centrifuged in DMEM for 5 min at 1500 rpm. The supernatant was removed, and the cells were suspended in DMEM medium. For sterilization of film samples, UV light (30 min exposure time) was employed. Then sterile samples were cultured in cell culture media (1.0 mL/cm^2^) for a day at 37°C with 5% CO_2_. The culture media with film extracts was combined with an equal volume of fresh media, filtered through a 0.22 µm syringe filter before experiments. Cells were placed at a concentration of 5 × 10^3^ cells per well in 96‐well plates with 100 µL of medium. The cells were cultivated in a CO_2_ incubator with 5.0% humidity at 37°C during hydrogel incubation. The cell culture media was replaced the following day with 100 µL of sample extract. Untreated cells were cultured in 100 µL of fresh media as a control. After 1st day, 10 µL of MTT solution (with a final concentration of 0.50 mg/mL) was introduced to each well, and cells were incubated for an additional 4.0 h. To dissolve the formazan crystals, the medium was discarded, and 100 µL of DMSO was introduced. The absorbance of the resulting solution was determined at 570 nm utilizing a microplate reader (SPECTROstar Nano Plate Reader, BMG LABTECH) after a final 20‐min incubation (Yaşayan et al. 2021; Alavarse et al. 2017).

#### 2.5.9 Scratch Assay

An in vitro scratch assay was employed to evaluate the wound healing performance of the hydrogel wound dressing films. NIH/3T3 cells were placed in 24‐well plates at a concentration of 25 × 10^3^ cells per well and permitted to achieve 90% confluence. A small scratch was created vertically in the cell monolayers using a 200 µL sterile pipette tip, mimicking a wound. PBS washing was used to eliminate any dislodged cells and debris. Film extracts were prepared following the MTT assay procedure, added to the wells, and incubated at 37°C with 5% CO_2_ in a 37°C incubator. A reverse microscope (Axiovert 25Carl Zeiss) was utilized to observe cell movement at the scratch location during designated time periods (0.0, 4.0, 8.0, 24, and 48 h) (Goetsch and Niesler 2011; Yaşayan et al. 2021).

#### 2.5.10 Antibacterial Test

Antimicrobial properties of the biogenic AgNPs were examined using the Kirby Bauer disc diffusion technique against clinically isolated *K. pneumoniae*, *C. Albicans*, and *B. subtilis* ATCC6633 according to Clinical and Laboratory Standards Institute Guidelines (Edition 2012). These species were supplied by Ege University. In summary, the bacteria were introduced into nutrient agar media and cultured for 24 h at 37°C in the incubator. After incubation, fresh colonies were used to prepare an inoculum at a 0.5 McFarland turbidity. The bacteria suspension was then spread across Muller Hinton agar plates using a sterile swap. Following the placement of a 6 mm sample‐impregnated disc, the plates were incubated for an additional 24 h. The antimicrobial effectiveness of the films was assessed by measuring the diameter of the inhibition zone. The provided data were studied in triplicates and the mean value of the results was calculated by standard error.

#### 2.5.11 Hemolysis Test

Hemolysis test was conducted on the hydrogel wound dressing films to assess their hemocompatibility, as severe lysis of red blood cells may signify potential harmful effects and restrict the safe application of the materials in contact with blood. First, entire bovine blood was gathered from the slaughterhouse and spun at 3500 rpm for 5 min in tubes containing anticoagulant. The red blood cells (RBCs) were rinsed three times using PBS after plasma removal. After the third rinse, RBCs were diluted in a 1:9 ratio with PBS. Film samples were subjected to a 2‐h incubation at 37°C alongside a 1:1 (v/v) RBC solution in PBS at varying concentrations (1‐15 g/mL). PBS and deionized water were used as negative and positive controls, respectively. Post‐incubation, the mixtures of samples and blood in microcentrifuge tubes were spun for 10 min at 3500 rpm After spinning, the tubes were carefully gathered without disrupting the pellets, photographed, and the supernatant from each tube was relocated to a microplate for optical density (OD) measurement using an ELISA reader (SPECTROstar Nano Plate Reader, BMG LABTECH). The subsequent equation was employed to calculate the percentage of hemolysis (Cao et al. 2021; Suneetha et al. 2019):

$$\text{Hemolysis}\;\%=\left[\;\frac{{\text{OD}}_{\text{sample}}-{\text{OD}}_{\text{negative}}}{{\text{OD}}_{\text{positive}}-{\text{OD}}_{\text{negative}}}\right]\times 100$$

Where OD_sample_ is the absorbance value of the test samples, OD_negative_ and OD_positive_ are the negative control (PBS) and positive control (deionized water), respectively.

### 2.6 Statistical Analysis

A minimum of three parallel studies was carried in each study. T‐test was performed for evaluation of the statistical analysis, and p values less than 0.05 were considered statistically significant (Yaşayan et al. 2021).

## 3 Results and Discussions

**Figure 1:**
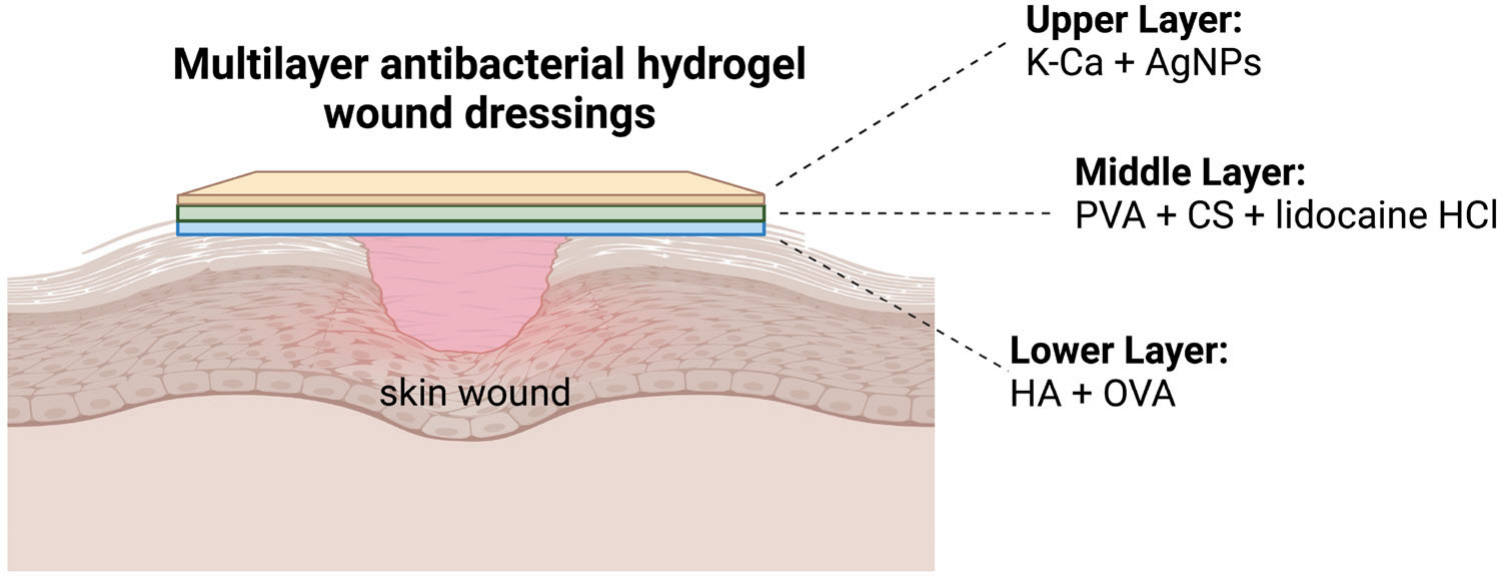
Schematic presentation of the multilayer wound dressings. The upper layer is composed of K‐ca and green synthesized AgNPs. Middle layer is composed of PVA, CS and lidocaine HCl. The lower layer is composed of HA and OVA.

Multilayer wound dressings were prepared as three layers by using solvent casting method (Figure 1). The upper layers (K‐ca and green synthesized AgNPs using apricot kernel skin extract) provide the moist control physical and barrier for microorganisms. AgNPs are utilized in wound dressings due to their wide‐ranging antibacterial action, superior effectiveness, and extended duration of activity (Zhang et al. 2023). They are a potential alternative to antibiotics due to their ability to kill various disease‐causing agents and the absence of antibiotic resistance induction (Krishnan et al. 2020). Besides antibacterial activity, recent research has demonstrated that AgNPs exhibit anti‐fungal, antiviral, anti‐inflammatory, and antiangiogenic properties, particularly notable for their antibacterial and anticancer capabilities (Yeşilot and Aydın Acar 2019). Moreover, several studies have suggested that AgNPs possess immunomodulatory properties, which might enhance their antibacterial effects by regulating inflammatory processes. In literature studies have shown that AgNPs reduce the inflammatory cytokines concentrations like interleukin (IL)‐1β, IL‐6, IL‐17, tumor necrosis factor alpha (TNF‐α) and transforming growth factor beta (TGF‐β) (Allawadhi et al. 2021). AgNPs have also been linked innate immune response, and reduce scar formation (Rybka et al. 2023). In this study, AgNPs are green synthesied from apricot kernel shell to eliminate use of hazardous chemicals, and costly and labor‐intensive production procedures (Moradi et al. 2021; Ratan et al. 2020). K‐ca is a hydrophilic colloid obtained from red marine algae like Kiringa, Stonecrop, and Deerstalker species. It consists of alternating d‐galactose and 3,6‐anhydrogalactose (3,6‐ag) units connected by ‐1,3 and ‐1,4 glycosidic bonds. They are classified as K‐type (Kappa), I‐type (Iota), and l‐type (Lambda) based on the types of sulfate binding present. K‐ca is extensively used in the pharmaceutical industry due to its gel‐forming, thickening, and emulsifying properties (Rahimi et al. 2020; Shen et al. 2021; Thomas et al. 2020). K‐ca based wound dressings have a high elasticity and stability, and this polymer can significantly boost wound dressing rigidity, flexibility, and capacity for retaining moisture (Shen et al. 2021; Alizadeh et al. 2023; Tavakoli et al. 2020).

Drug‐loaded PVA and CS‐based middle layer was designed as a layer capable of controlled drug release and to add strength to the structure. Lidocaine HCl was used as model drug. CS is a natural polysaccharide composed of d‐glucosamine and n‐acetylglucosamine that is generated via acetylation of chitin. This polymer has antibacterial and anti‐inflammatory properties. CS is a commonly used substance in biomedical applications, particularly in wound dressings due to its water‐absorbing capability, high biocompatibility, low toxicity, and immune‐stimulatory effects (Rahimi et al. 2020; Shen et al. 2021; Hamedi et al. 2022; Yang et al. 2020). Moreover, it is an effective natural blood‐clotting agent, promoting red blood cell clustering and tissue repair (Zhang et al. 2023). CS reduces the formation of scars, and take a role in scar tissue regeneration and re‐epithelialization (Shen et al. 2021; Alven and Aderibigbe 2021; Valachová et al. 2022). PVA is a widely used synthetic polymer made up of vinyl acetate monomer and hydrolyzed acetate. Its popularity in fabrication of wound dressings can be attributed to its biocompatibility, biodegradability, hydrophilicity, bio‐adhesiveness, nontoxic nature, and chemical resistance (Amer et al. 2022; Kamoun et al. 2021).

The lower layer (HA and OVA) serves as the controlling membrane for controlled drug release and wound healing. HA is a naturally occurring polyanionic glycosaminoglycan that plays a significant function in the ECM. It consists of two recurring linear disaccharides, d‐glucuronic acid and 2‐acetamido‐2‐deoxy‐d‐glucopyranose. Due to its exceptional water solubility, HA produces thick solutions with remarkable viscoelastic features. The internal hydrogen bonds in HA chains construct a 3D configuration that has the capacity to hold nearly 1000 times its own mass in water (Valachová et al. 2022; Castro et al. 2021; Graça et al. 2020). HA contributes to all stages of the wound healing process, as well as participating in tissue restructuring and the innate immune reaction to tissue injury; the biological characteristics of HA encompass cellular attachment, growth, movement, development of granulation tissue, and predominant presence in connective tissue structures (Shen et al. 2021; Valachová et al. 2022). OVA consists of 386 amino acids and is accounting for 54% of the total egg white protein content. OVA has good gelling, foaming, and emulsifying capabilities. OVA binds to the wound and promotes the regeneration of damaged skin tissue. It has been demonstrated the ability to enhance cell adhesion and growth and it is suitable for uses as a delivery system (Zhang et al. 2023; Guo et al. 2022). Besides these, it is an easily accessible and cost‐effective protein option (Guo et al. 2022).

### 3.1 Size and Zeta Potential Measurement Results of AgNPs

A biogenic synthesis method was used for synthesis of the AgNPs from apricot kernel skin extract (Nejati. 2024). After synthesis of the AgNPs, DLS and zeta potential measurements of the AgNPs were performed. The size and zeta potential of AgNPs are presented in Figure 2. The particle size of the AgNPs were found to be 121.7 nm with a dispersion index of 0.394. DLS results indicated that AgNPs are in nano‐diameters, and according to PDI values, size distributions with a moderate width are obtained (Pistone et al. 2016). Zeta potential of AgNPs were measured as −35.3 mV, indicating high stability of the particles due to avoidance of aggregation due to high charge on the surface of the nanoparticles (Pistone et al. 2016).

**Figure ‎2:**
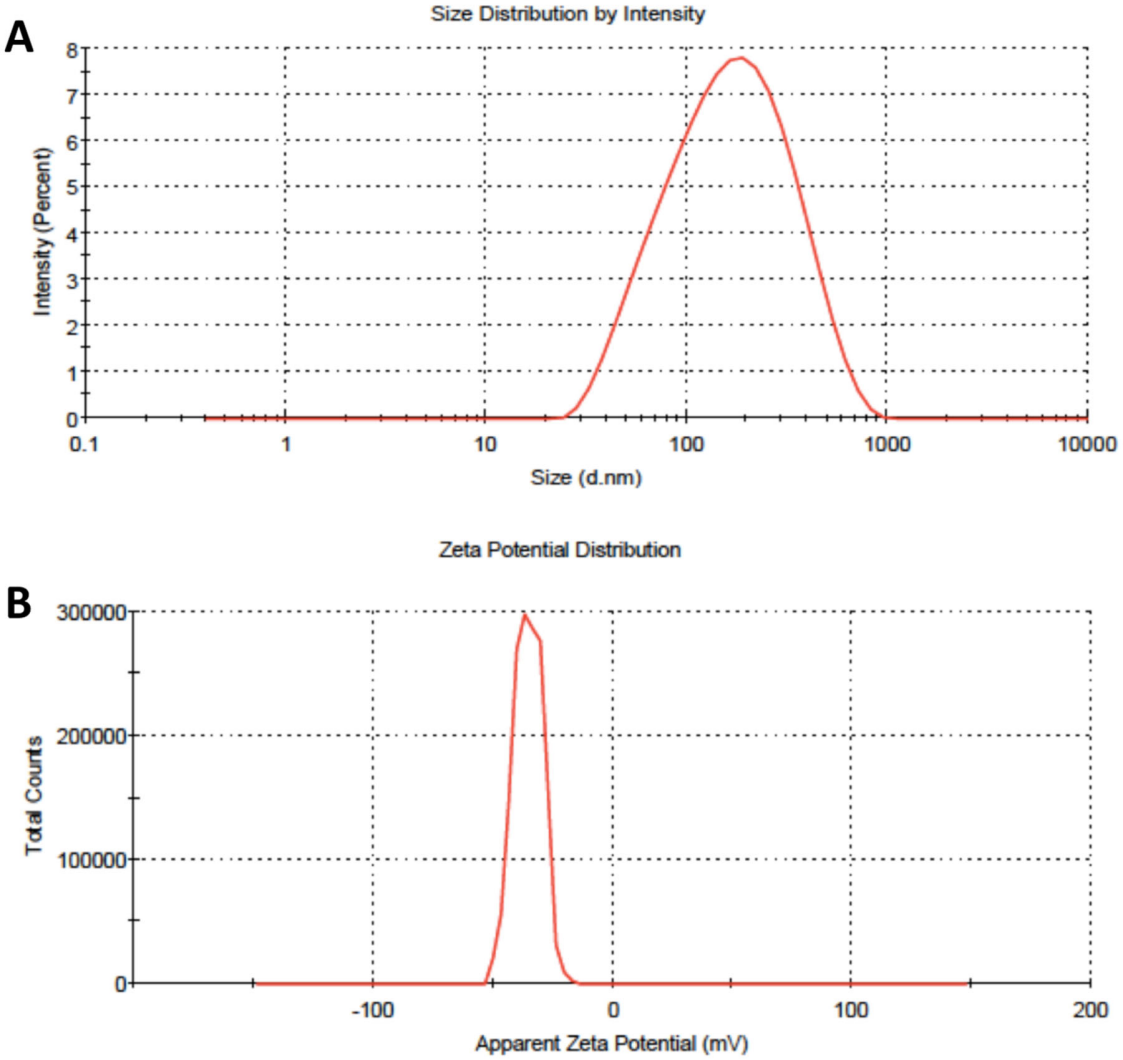
Dynamic light scattering (A) and zeta potential measurement (B) results of AgNPs.

### 3.2 SDS‐PAGE Results

OVA is synthesized as described in the Section 2.3. Following the synthesis, an SDS‐PAGE analysis was carried for conformation of the polymer. The SDS‐PAGE results and Coomassie staining indicate that the expected bands of OVA (45 kDa) were observed (Figure S1), which confirms that the extraction procedure of OVA from egg white has been done correctly (Shojaee et al. 2015).

### 3.3 FTIR Results

**Figure 3:**
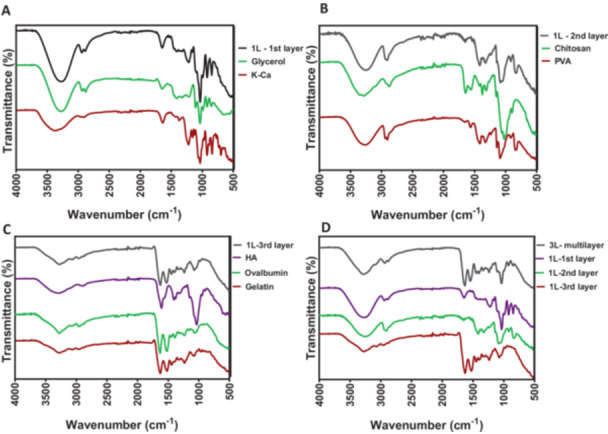
FTIR results of hydrogel wound dressing films: (A) lower layer of the monolayer films and its polymeric content, (B) middle layer of the monolayer films and its polymeric content, (C) lower layer of the monolayer films and its polymeric content, (D) 3‐layered multilayer film and monolayer films.

The upper first layer of the dressings is composed of K‐ca and AgNPs, while middle (second) layer includes PVA, CS, and the lower (third) layer is composed of HA acid, OVA and gelatin. The FTIR analysis of wound dressing samples and the ingredients alone was conducted to evaluate compatibility of formulation ingredients. The FTIR results are shown in Figure 3. Figure 3A shows the main bands of the polymers K‐ca and glycerol separately, and in combination within the monolayer film. The upper layer of the film is named as “1L‐1st Layer.” In the FTIR spectra, the major peaks are found to be related to the hydroxyl and acetate groups as follows: The broad bands detected between 3550 and 3200 cm^−1^ are due to by O‐H stretching by intermolecular hydrogen bonding. The vibrational band identified between 2840 and 3000 cm^−1^ refers to C‐H stretching of alkyl groups, and the peaks between 1735 and 1750 cm^−1^ refer to C‐O stretching of acetate groups. Figure 3B shows the main bands of the polymers CS and PVA separately, and in combination within the monolayer film. The middle layer of the film is named as “1L‐2nd Layer.” The major peaks related to the hydroxyl and acetate groups were identified in the FTIR spectra as follows: The broad bands detected between 3550 and 3200 cm^−1^ are correspond to O‐H stretching by intermolecular hydrogen bonding. The vibrational band identified between 2840 and 3000 cm^−1^ refer to C‐H stretching of alkyl groups, while peaks between 1735 and 1750 cm^−1^ is due to C‐O stretching of acetate groups. Figure 3C shows the main bands of the polymers HA, OVA and gelatin separately, and in combination within the within the monolayer film. The lower layer of the film is named as “1L‐3rd Layer.” The major peaks identified in the FTIR spectra were related to the hydroxyl and acetate groups, and are given as follows: The broad bands detected between 3550 and 3200 cm^−1^ are correspond to O‐H stretching by intermolecular hydrogen bonding. The vibrational band identified between 2840 and 3000 cm^−1^ refers to C‐H stretching of alkyl groups, while peaks between 1735 and 1750 cm^−1^ correspond to C‐O stretching of acetate groups (Mansur et al. 2008).

### 3.4 Swelling Results

**Figure 4:**
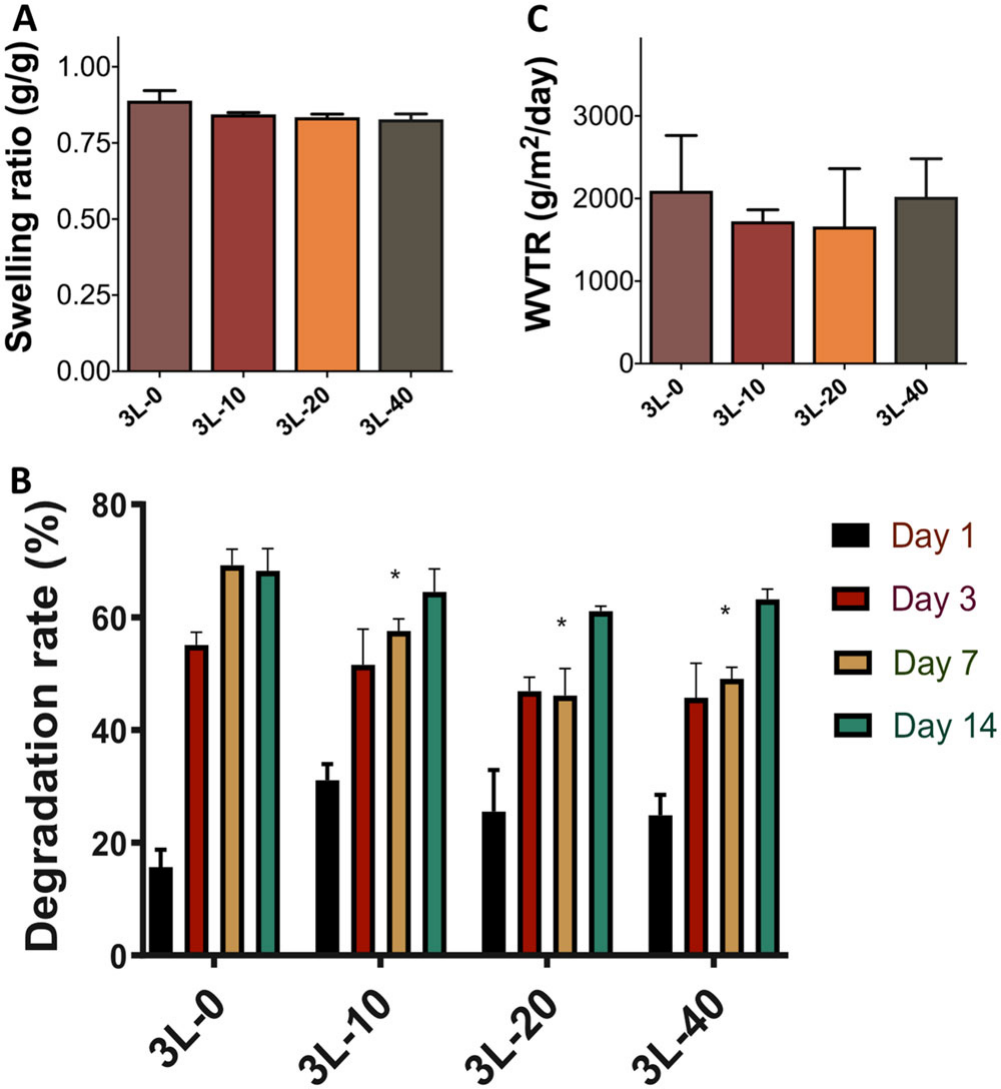
(A) Swelling ratio, (B) in vitro degradation rate and (C) water vapor permeability results of multilayer hydrogel wound dressings. 3L‐0 sample (sample without AgNPs) was compared with nanoparticle‐containing samples in each day (*P **≤** 0.05).

Swelling studies of hydrogel wound dressing were undertaken to measure the water uptake capability of the films, and to evaluate the effect of AgNPs encapsulating dressings in the film swelling. The swelling ratios for the 3L‐0, 3L‐10, 3L‐20, and 3L‐40 samples, as given in Figure 4A, were determined to be 0.89  ±  0.03 g/g, 0.84  ±  0.01 g/g, 0.83  ±  0.01 g/g, and 0.83  ±  0.02 g/g, respectively. The presence of AgNPs in the films encapsulating different ratios of AgNPs (0, 10, 20, and 40 µg/mL) have no significant effect statistically on the swelling ratio of the samples compared to the samples in the control group.

### 3.5 In Vitro Degradation Results

Stability of the wound dressing is another critical parameter; dressings should be durable to avoid frequent changes not disintegrate too quickly for wound dressing applications (Op't Veld et al. 2020). To evaluate in vitro degradation behavior of the samples, multilayer wound dressing films were incubated in PBS at 37°C for 14 days. According to the results given in Figure 4B, all of the hydrogels degrade slowly after 14 days. The addition of AgNPs in the formulation increase the hydrogel degradation rate on 1st day, and this could be attributed to burst release of the AgNPs from the dressing, that may result in a decrease on sample weight compared to 3L‐0 samples. However, no statistical significance was calculated when 3L‐0 sample (sample without nanoparticle) was compared with nanoparticle‐containing samples. In the 3rd day, faster degradation of the 3L‐0 sample was observed compared to the samples containing nanoparticles, however, no statistical significance was calculated again when 3L‐0 sample was compared to nanoparticle containing samples. For day 7, degradation rate of 3L‐0 sample was statistically significant compared to 3L‐10, 3L‐20 and 3L‐40 samples. As AgNPs may affect both chemical and physical properties of the samples such like porosity, morphology, this change is attributed to influence of AgNPs (Zhao et al. 2020). As shown in Figure 4B, the degradation rates at day 14 for the 3L‐0, 3L‐10, 3L‐20, and 3L‐40 samples were determined to be 68.32% ± 7.66%, 64.55% ± 8.12%, 61.11% ± 1.83%, and 63.22% ± 3.70%, respectively. These results indicate that all groups exhibited comparable degradation rates by Day 14.

### 3.6 Water Vapor Permeability Results and Physical Examination of the Samples

Maintaining a moist environment has been demonstrated to promote wound healing by preventing dehydration and boosting both angiogenesis and collagen synthesis. Additionally, it facilitates the increased breakdown of dead tissue and fibrin, ultimately enhancing the overall appearance of the wound and reducing pain (Junker et al. 2013). Therefore prevention and management of epidermal water loss from the wound are critical for faster and more successful wound healing (Türkoğlu et al. 2021). To evaluate water loss from the wound area after placement of the dressings, WVTR of the samples were measured. The WVTR values of multi‐layered hydrogel wound dressing are given in Figure 4C. According to results, the water vapor permeability from the samples did not cause a significant difference between the AgNPs‐containing dressings and the control sample (*p* > 0.05). In the literature, a WVTR of 2000 to 2500 g/m^2^ in 24 h is acceptable (Demir et al. 2022), and our results demonstrate that the WVTR range is 2096 ± 666 g/m^2^ for 3L‐0 sample, 1726 ± 139 g/m^2^ for 3L‐10 sample, 1661 ± 670 g/m^2^ for 3L‐20 sample and 2022 ± 460 g/m^2^ for 3L‐40 sample, which indicates that all wound dressing samples have acceptable WVTR values to be used as wound dressings.

The mass per unit area, thickness, and pH examination findings of multilayer hydrogel dressings were illustrated in Table S1. It was observed that the samples become bulky depending on the increasing concentrations of AgNPs. In pH measurements, it was observed that the pH value was increased slightly, from neutral to alkaline in all samples.

### 3.7 Mechanical Test Results

**Figure 5:**
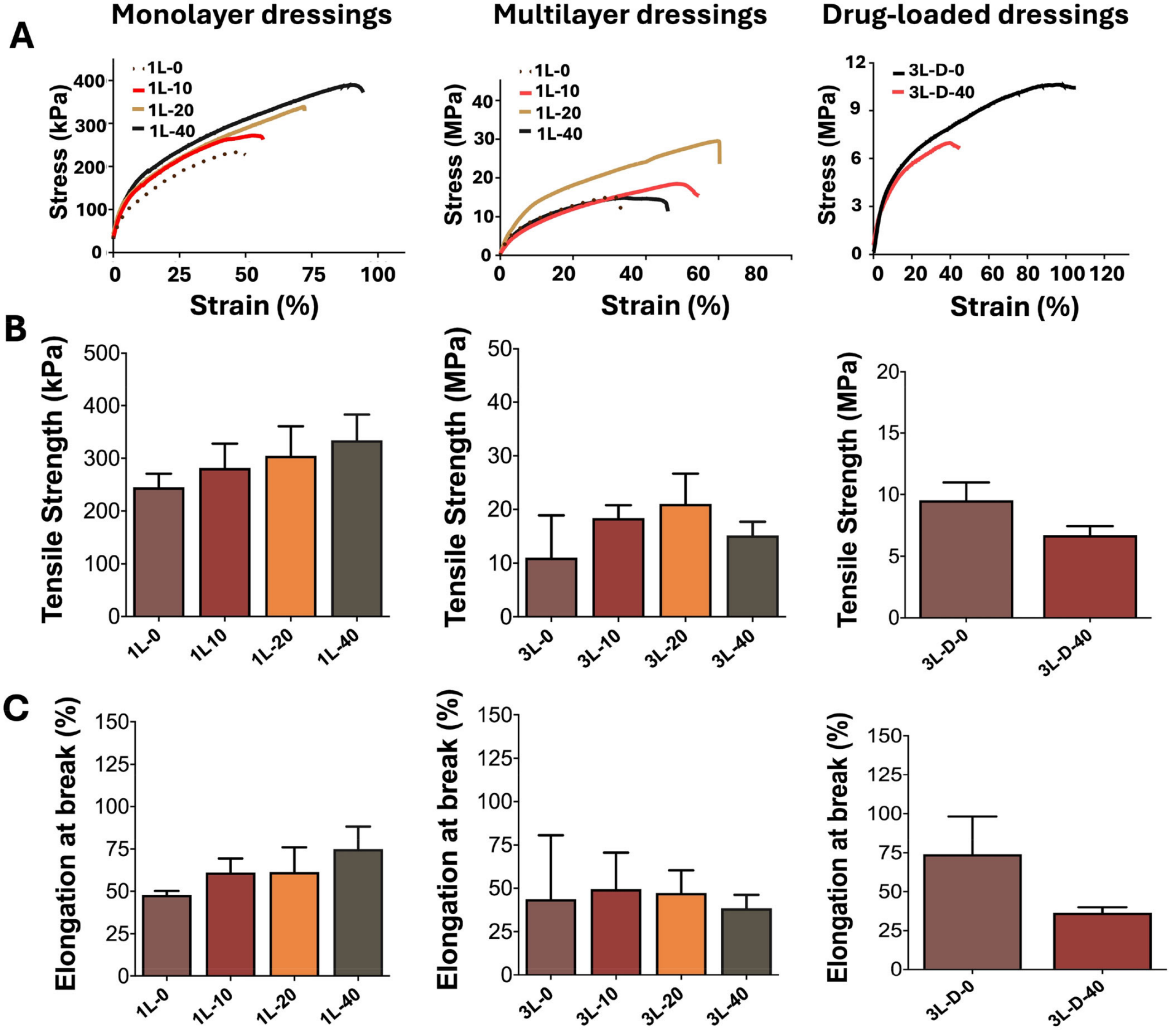
Mechanical properties of monolayer, multilayer and lidocaine HCl‐loaded hydrogel wound dressings: (A) stress‐strain curves, (B) tensile strength and (C) elongation at break.

Mechanical tests of monolayer, multilayer and drug‐loaded hydrogel wound dressing films were carried, and the results are given in Figure 5. Tensile strength tests on hydrogel films were performed on monolayer samples without (1L‐0) and with AgNPs (1L‐10, 1L‐20 1L‐40). The samples have a tensile strength of roughly around 200‐400 kPa. According to results, both the elongation at break and the tensile strength showed an increase with an increase in the amount of AgNPs in the formulation. The elongation at break increased from 47.99% ± 2.28% (1L‐0) to 74.99% ± 13.24% (1L‐40) by increase in the AgNPs amounts. Tensile strength tests on multilayer hydrogel films were also performed on samples without (3L‐0) and with AgNPs (3L‐10, 3L‐20 3L‐40). According to stress‐strain curves of samples, the 3L‐20 sample have showed improved properties compared to the other groups. This data indicates that concentration of AgNPs in samples have significant importance in increasing the resistance of the layers. No significant difference was observed in the elongation values at break of the samples. Mechanical behaviors of lidocaine HCl‐loaded multilayer hydrogel films without (3L‐D‐0) and with AgNPs (3L‐D‐40) were investigated as well. The results indicate that addition of lidocaine HCl to the structure resulted a decrease in the mechanical properties of the 3L‐D‐40 multilayer films. The tensile strength decreased from 9.53 ± 1.46 MPa to 6.71 ± 0.72 MPa, and the elongation at break decreased from 74.05% ± 24.13% to 36.38% ± 3.62% after addition of the drug. It is known that drug–polymer interactions could result in significant impacts on mechanical properties of the samples, and depending on the chemical properties of the drug and its amount within the sample, mechanical properties of the sample could vary significantly (Chou and Woodrow 2017).

### 3.8 Drug Release Profile

**Figure 6:**
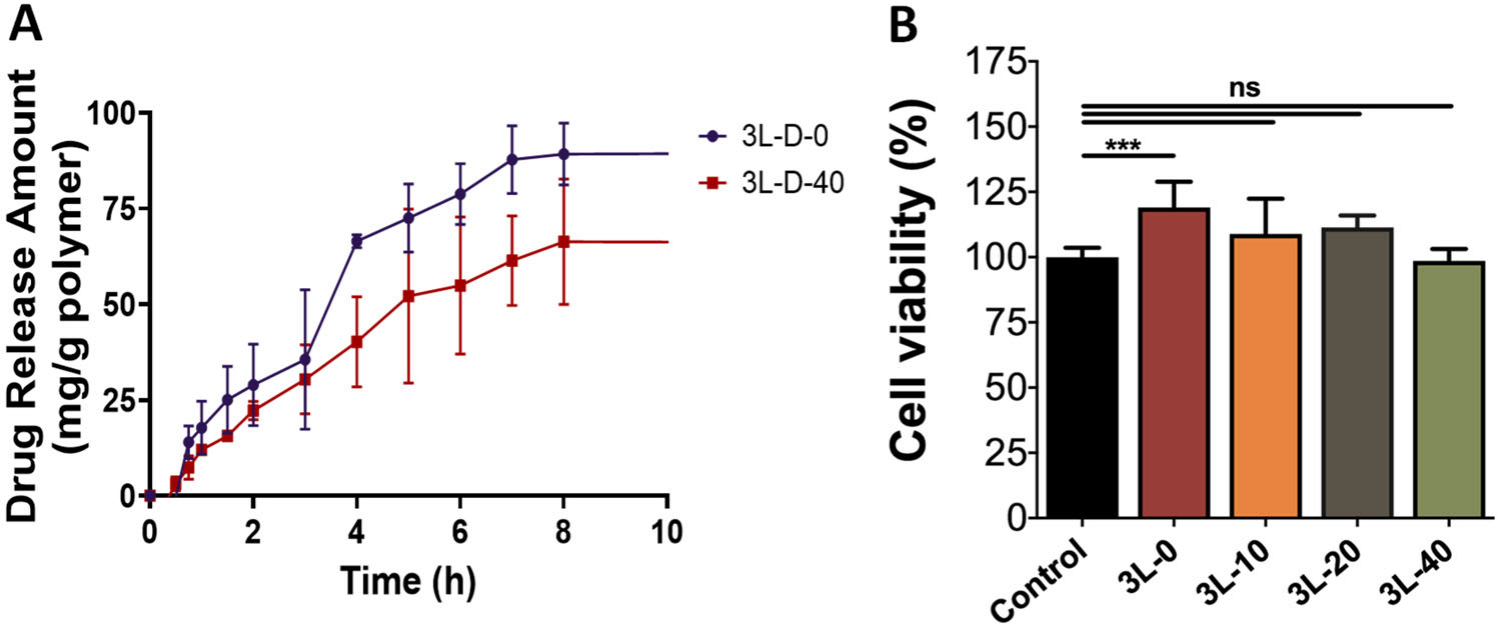
(A) In vitro cumulative lidocaine HCl release profile for 3L‐D‐0 and 3L‐D‐40 multi‐ layer hydrogel wound dressings. (B) In vitro cytotoxicity behavior of the multilayer hydrogel wound dressings. 3L‐0, 3L‐10, 3L‐20 and 3L‐40 for multilayer hydrogels were used during the studies. Untreated cells were cultured as a control. The study is conducted on NIH/3T3 cell line (ns: *P* > 0.05 and ***: P ≤ 0.001).

The cumulative drug release properties of lidocaine HCl from multi‐layer hydrogel wound dressings was performed. Samples with and without AgNPs were studied in drug release studies. In the studies, 3L‐D‐0, and 3L‐D‐40 were examined, and drug release was quantified using a UV‐Vis spectrophotometer at time intervals to 10 h. The results are given in Figure 6A. Lidocaine HCl was selected as a model drug due to anesthetic properties for reducing pain and suffering caused by injuries, providing relief to the patient. According to obtained results, addition of nanoparticles affects cumulative release of the drug from the formulations, and the formulations with AgNPs have an extended‐release rate compared to formulation without nanoparticles. In overall evaluation of drug release, 90.00% ± 4.39% of the drug was released from 3L‐D‐0 samples, and 66.72% ± 14.79% of drug release from 3L‐D‐40 samples at the end of 10 h. According to release kinetics plots, R^2^ values for 3L‐D‐0 samples are calculated as 0.8743, 0.8992, 0.7461, and 0.8916 for zero‐order, first‐order, Higuchi, and Hixson Crowell release model, respectively. For 3L‐D‐40 samples, R^2^ values are 0.9471, 0.9549, 0.8013, and 0.953 for zero‐order, first‐order, Higuchi, and Hixson Crowell release model, respectively. According R^2^ values, drug release fits to first‐order model for both 3L‐D‐0 and 3L‐D‐40 samples, which indicates that drug release mechanism is concentration dependent and sustained release is achieved from the multilayer films (Shafiei et al. 2021).

### 3.9 Cell Viability Results

The MTT assay (3‐[4, 5‐dimethylthiazol‐2‐yl]‐2, 5 diphenyl tetrazolium bromide) assesses mitochondrial function and estimates the metabolic activity of viable cells by transforming MTT into formazan crystals within living cells (van Meerloo et al. 2011). This assay relies on the enzymatic reduction of the light‐colored tetrazolium salt to its deep blue‐violet formazan counterpart, which can be quantified using spectrophotometry (Grela et al. 2018). The overall mitochondrial activity is generally proportional to the number of living cells within the majority of cell populations; therefore, this method is commonly employed to assess the cytotoxic impacts of pharmaceuticals on both cell lines and primary cells from patients (van Meerloo et al. 2011; Ghasemi et al. 2021).

The toxicity of hydrogel wound dressing films including green biosynthesized AgNPs in various amounts was examined using the MTT test against NIH/3T3 cell lines. The NIH/3T3 cell viability percentage after 24 h of treatment was measured. MTT tests results of multilayer hydrogel wound dressing films were given in Figure 6B. The cell viability percentages in all samples were higher compared to the control sample except the 3L‐40. 3L‐40 found to have a similar viability compared to the control sample (98.56% ± 4.58%). The results indicate that the wound dressing samples are not cytotoxic. Among the samples, AgNPs‐free hydrogel films supported cell viability greater than hydrogel films containing AgNPs (Khan et al. 2021).

### 3.10 In Vitro Scratch Assay Results

**Figure 7:**
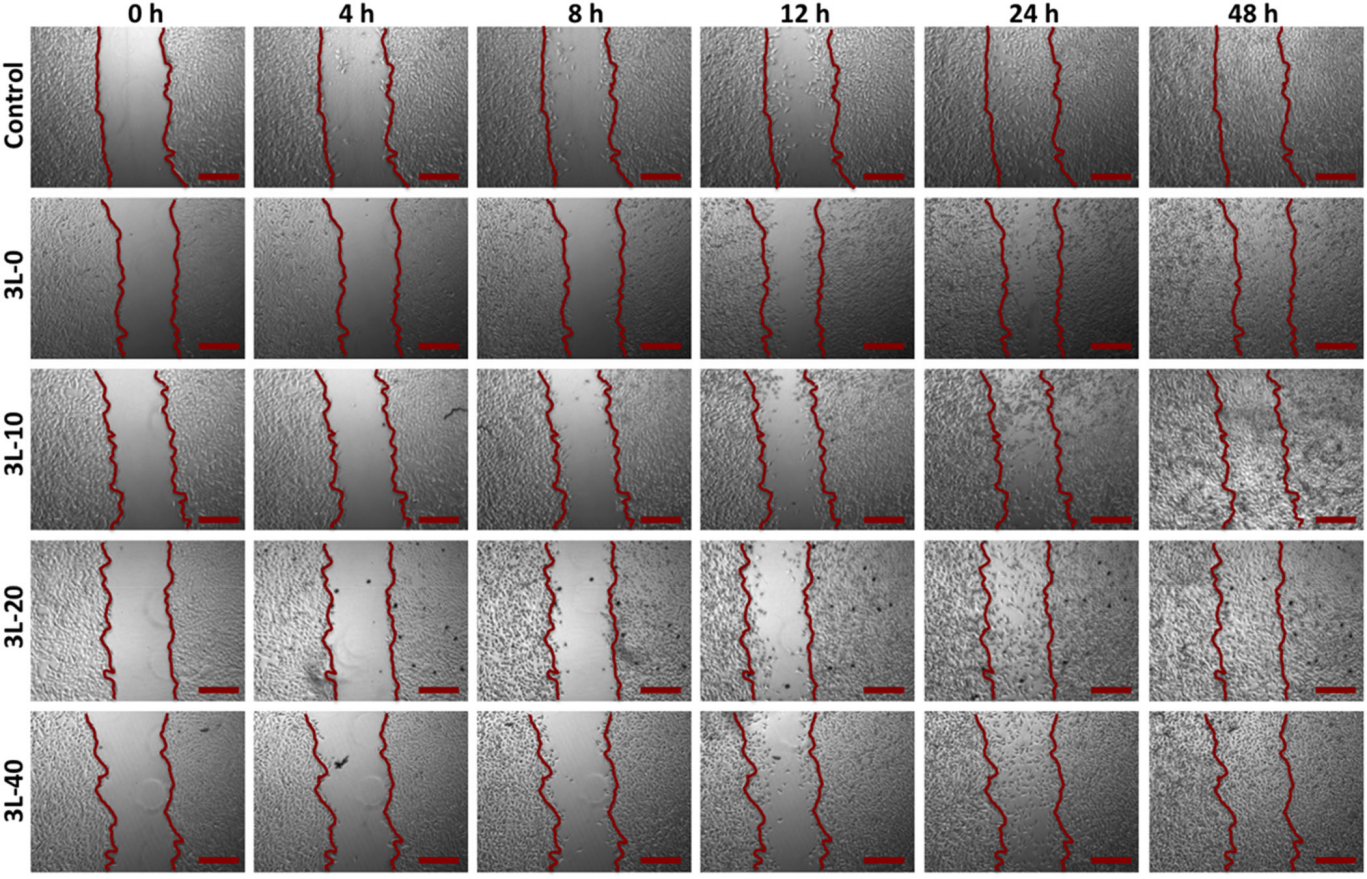
In vitro scratch assay of the multilayer hydrogel wound dressings at 0, 4, 8, 12, 24 and 48 h. Untreated cells were cultured as a control. The study was conducted on NIH/3T3 cell line (Scale bar: 250 um).

Fibroblast cell migration is another critical parameter in wound healing, an in vitro scratch test was carried to assess the effects of varying concentrations of AgNPs in the films on the migratory capacity of NIH/3T3 cells. The scratch region did not demonstrate a substantial increase in cell migration against the 3T3 cell line after 48 h of incubation, as shown in Figure 7. In a study in the literature, AgNPs produced using *Heliotropium bacciferum* plant extract were also demonstrated to impede cell migration in a study done by Khan et al (Khan et al. 2021)., which demonstrates that the obtained data is consistent with the literature, and all films are biocompatible, and non‐cytotoxic for use in wound healing applications.

### 3.11 Antibacterial Test Results

Antibacterial activity tests of multilayer hydrogel wound dressing films were performed to evaluate antimicrobial properties of AgNPs. It is demonstrated in literature that AgNPs have the ability to connect to the bacterial cell wall and disrupt the cell's integrity by releasing silver ions (More et al. 2023). AgNPs also interfere with DNA replication in bacteria (Yang et al. 2009). AgNPs’ reduced size also plays a significant part in the process of antibacterial potential, allowing them to efficiently overcome membrane barriers and damage the physical and chemical activities of the bacterium (Bhat et al. 2021; Selvam et al. 2017).

**Figure 8:**
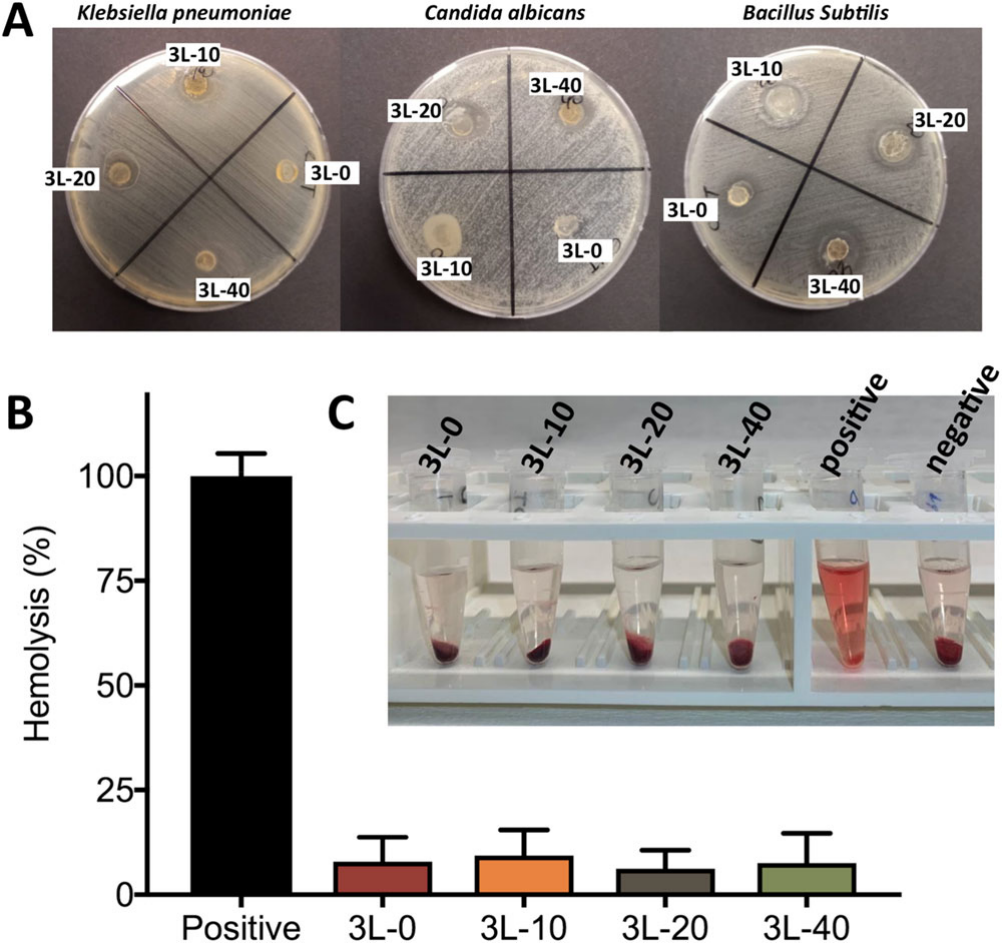
(A) Antibacterial potential of multilayer wound dressing films against *Klebsiella Pneumoniae*, *Candida Albicans* and *Bacillus Subtilis*. 3L‐0, 3L‐10, 3L‐20 and 3L‐40 for multilayer hydrogels were used during the studies. (B) Hemolysis (%) results and (C) photographic images of hemolysis test demonstrating hemolysis results of multilayer hydrogel wound dressings. Deionized water and PBS were used as the positive and negative controls, respectively.

In this study, the antibacterial efficacy of Biogen AgNPs against a Gram‐negative bacteria (*K. pneumoniae*), a Gram‐positive bacterium (*B. subtilis*) as well as *C. albicans* were investigated in this study. During the studies, films including three different amounts of AgNPs were evaluated (3L‐0, 3L‐10, 3L‐20 and 3L‐40). According to results, biogenic AgNPs had an antibacterial effect on all three types of microorganisms, and the antibacterial efficacy of films with AgNPs was found to follow a dose‐dependent activity against *K. pneumoniae*, *B. subtilis*, and *C. albicans* (Figure 8A and Table S2). In 3L‐40 sample film, the maximum antibacterial potential was observed against *B. subtilis* (zone diameter: 17 ± 1.2 mm) and *C. albicans* (zone diameter: 10 ± 0.6 mm). 3L‐20 sample film showed best antimicrobial activity against *K. pneumoniae* (zone diameter: 5 ± 4 mm).

Several investigations have demonstrated that AgNPs have a biocidal effect on all Gram‐positive and Gram‐negative bacteria. Selvam et al. evaluated the antibacterial activity of biosynthesized AgNPs with *Tinospora cordifolia* (*Thunb.)* against *Staphylococcus sp*., *Klebsiella sp*., and *Bacillus sp*., just one concentration (10 ug/mL) and they found efficacy against *S. aureus* at a zone diameter of 10 mm, *Klebsiella* at a zone diamater of 12.3 mm, and *B. subtililis at* a 10.5 mm (Selvam et al. 2017). Comparing to our results, they found more effectiveness at low concentration by using *Tinospora cordifolia* extract and in this study apricot carnel was used as a reducing agent diversely, and we noticed that as the concentration increased, the antimicrobial activity increased too, and most effective result was obtained against *B. subtilis* (inhibition zone = 17 + 1.2 at 40 ug/mL concentration) (Selvam et al. 2017). In another research, Bayav et al. searched the antimicrobial effectiveness of apricot kernel AgNPs and they got results that support our study but with less effectiveness (Bayav et al. 2024). Considering the studies conducted to date, the use of apricot kernel as a reducing agent in the synthesis of AgNPs has not been detailly assessed.

### 3.12 Hemolysis Test Results

Hemolysis tests are being conducted to better understand compatibility of the wound dressings with RBCs (Chen et al. 2015). Many therapeutic compounds can interact with RBCs once they enter the bloodstream. It has recently been demonstrated that AgNPs can cause membrane damage and lysis of RBCs via an oxidative stress mechanism (Nasar et al. 2022). The degree of damage is proportional to the size of the AgNPs, and smaller AgNPs found to have a greater hemolytic capability (Nasar et al. 2022; Dudhagara et al. 2022). Hemolysis tests of multilayer hydrogel wound dressing films were performed with samples 3L‐0, 3L‐10, 3L‐20 and 3L‐40, and the findings of the hemolytic activity assay are given in Figure 8B,C. During the studies, deionized water as the positive control, while PBS acted as the negative control. According to results, all the wound dressing formulations encapsulating AgNPs were shown to be safe, causing no substantial damage to red blood cells and not lysing them.

## 4 Conclusion

In recent years, wound management, treatment, and recovery processes have evolved, and significant progress has been made in this area particularly by introducing novel components in wound dressings including various types of nanoparticles and biomaterials. It is found that novel hydrogel wound dressings could be more useful compared to traditional dressings to reduce the incidence of infection and mortality rate in patients with all types of wounds. Despite these progresses, wound infection remains one of the leading causes of impaired healing, and may lead to death, especially chronic wounds and burns. To design a wound dressing for infected wounds, we have developed novel multilayered antibacterial hydrogel wound dressings encapsulating AgNPs and lidocaine HCl. Biogenic nanoparticles were synthesized to reduce the cytotoxicity of chemicals. According to our findings, the use of AgNPs have improved the stability and strength of the hydrogel by increasing the control of moisture and water vapor retention in the wound, keeping factors like swelling and degradation rates stable, and maintaining the continuous and incremental release of the drug. The polymeric components in this multilayered hydrogel, such as CS, HA, and K‐ca, found to improve adherence, and aid wound healing. The dressings did not exhibit cytotoxicity against fibroblasts. The findings of bacterial zone of inhibition test against *K. pneumoniae*, *B. subtilis*, and *C. albicans* demonstrated that the presence of AgNPs enable antimicrobial property of the dressing. In conclusion, the design and formulation of the multilayered hydrogel exhibit optimal qualities to facilitate the wound healing process. The optimized multilayer hydrogel film, containing AgNPs and lidocaine HCl, demonstrated excellent antibacterial potential, biocompatibility and hemocompatibility, with approximately 60% degradation by day 14, a water vapor permeability of 2022 ±  460 g/m²/24 h, a tensile strength of 6.71 ± 0.62 MPa, an elongation at break of 36.38% ± 3.62%, and a cumulative drug release of 65.72% ± 14.80% within 10 h. These attributes jointly enhance its viability as an efficacious wound dressing material.

## Conflicts of Interest

The authors declare no conflicts of interest.

## Supporting information

Supporting Information.

## Data Availability

The data that support the findings of this study are available from the corresponding author upon reasonable request.
