# Supplementary material for: Multilayer Antibacterial Hydrogel Wound Dressings Incorporated With Green Synthesized Silver Nanoparticles
Source: Drug Dev Res. 2025 May 8;86(3):e70102. doi: 10.1002/ddr.70102 (PMC12060213; doi:10.1002/ddr.70102)
Supplement: Supplementary file 1 — Supporting Information. [file DDR-86-e70102-s001.docx]

Supporting Information

**Multilayer antibacterial hydrogel wound dressings incorporated with green synthesized silver nanoparticles**

Ali Alipour^1^, Omid Nejati^1^, Gökçen Yaşayan^2^, Ayça Girgin^3^, Buse Tuğba Zaman^3^, Betül Giray^4^, Okşan Karal-Yılmaz^5^, Sezgin Bakırdere^3,6^, Ayça Bal-Öztürk^1,7,8,*^

^1^ Department of Stem Cell and Tissue Engineering, Institute of Graduate Education, İstinye University, 34010, İstanbul, Türkiye

^2^Department of Pharmaceutical Technology, Faculty of Pharmacy, Yeditepe University, 34755, İstanbul, Türkiye

^3^ Chemistry Department, Faculty of Art and Science, Yıldız Technical University, 34210, İstanbul, Türkiye

^4^ Department of Pharmaceutical Microbiology, Faculty of Pharmacy, İstinye University, 34010, İstanbul, Türkiye

^5^ Department of Chemical Engineering, Faculty of Engineering and Architecture, İstanbul Beykent University, Sariyer, İstanbul 34398, Turkey

^6^ Turkish Academy of Sciences (TÜBA), 06670, Ankara, Türkiye

^7^ Department of Analytical Chemistry, Faculty of Pharmacy, İstinye University, 34010, İstanbul, Türkiye

^8^ Stem Cell and Tissue Engineering Application and Research Center (ISUKOK), İstinye University, 34010 İstanbul, Türkiye

*Corresponding authors: Ayça Bal-Öztürk: aycabal@gmail.com, aozturk@istinye.edu.tr


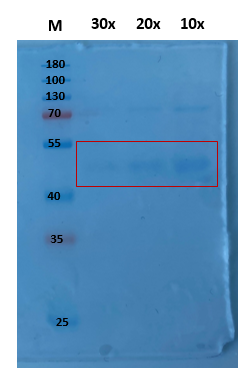


**Figure S1.** SDS-PAGE analysis results of OVA

**Table S1.** Physical properties (masses and thickness measurements) and pH values of multilayer hydrogel wound dressings.

| **Samples** | **Mass**  **(mg/cm^2^)** | **Thickness (mm)** | **pH** | |
| --- | --- | --- | --- | --- |
|  |  |  | **2 hours** | **24 hours** |
| 3L-0 | 52.00 ± 1.700 | 0.390 ± 0.006 | 5.38 ± 0.01 | 6.75 ± 0.54 |
| 3L-10 | 46.05 ± 0.640 | 0.365± 0.005 | 5.71 ± 0.01 | 6.75 ± 0.27 |
| 3L-20 | 50.40 ± 0.141 | 0.403 ± 0.010 | 5.90 ± 0.28 | 6.80 ± 0.04 |
| 3L-40 | 45.95 ± 0.495 | 0.410 ± 0.006 | 6.15 ± 0.35 | 6.77 ± 0.16 |

**Table S2:** Antimicrobial Activity of the multilayer wound dressing films in different concentrations against *Klebsiella pneumoniae*, *Bacillus subtilis* and *Candida albicans.*

| **Samples** | ***Klebsiella pneumonia***  **(zone diameter, mm)** | ***Candida albicans***  **(zone diameter, mm)** | **Bacillus subtilis ATCC6633**  **(zone diameter, mm)** |
| --- | --- | --- | --- |
| 3L-0 | - | - | 7 ± 0.6 |
| 3L-10 | 7 ± 0.6 | - | 8 ± 0.6 |
| 3L-20 | 9 ± 1.5 | 8 ± 0.6 | 13 ± 1.2 |
| 3L-40 | 5 ± 4 | 10 ± 0.6 | 17 ± 1.2 |

* ’’-‘’ implies no inhibition at the studied concentration.
